# Species Used for Drug Testing Reveal Different Inhibition Susceptibility for 17beta-Hydroxysteroid Dehydrogenase Type 1

**DOI:** 10.1371/journal.pone.0010969

**Published:** 2010-06-08

**Authors:** Gabriele Möller, Bettina Husen, Dorota Kowalik, Leena Hirvelä, Dariusz Plewczynski, Leszek Rychlewski, Josef Messinger, Hubert Thole, Jerzy Adamski

**Affiliations:** 1 Helmholtz Zentrum München, Institute of Experimental Genetics, Genome Analysis Center, Neuherberg, Germany; 2 Solvay Pharmaceuticals Research Laboratories, Hannover, Germany; 3 Hormos Medical, Turku, Finland; 4 Interdisciplinary Centre for Mathematical and Computational Modelling, Warsaw University, Warsaw, Poland; 5 Bioinfobank Institute, Poznań, Poland; 6 Lehrstuhl für Experimentelle Genetik, Technische Universität München, Freising-Weihenstephan, Germany; Dr. Margarete Fischer-Bosch Institute of Clinical Pharmacology, Germany

## Abstract

Steroid-related cancers can be treated by inhibitors of steroid metabolism. In searching for new inhibitors of human 17beta-hydroxysteroid dehydrogenase type 1 (17β-HSD 1) for the treatment of breast cancer or endometriosis, novel substances based on 15-substituted estrone were validated. We checked the specificity for different 17β-HSD types and species. Compounds were tested for specificity *in vitro* not only towards recombinant human 17β-HSD types 1, 2, 4, 5 and 7 but also against 17β-HSD 1 of several other species including marmoset, pig, mouse, and rat. The latter are used in the processes of pharmacophore screening. We present the quantification of inhibitor preferences between human and animal models. Profound differences in the susceptibility to inhibition of steroid conversion among all 17β-HSDs analyzed were observed. Especially, the rodent 17β-HSDs 1 were significantly less sensitive to inhibition compared to the human ortholog, while the most similar inhibition pattern to the human 17β-HSD 1 was obtained with the marmoset enzyme. Molecular docking experiments predicted estrone as the most potent inhibitor. The best performing compound in enzymatic assays was also highly ranked by docking scoring for the human enzyme. However, species-specific prediction of inhibitor performance by molecular docking was not possible. We show that experiments with good candidate compounds would out-select them in the rodent model during preclinical optimization steps. Potentially active human-relevant drugs, therefore, would no longer be further developed. Activity and efficacy screens in heterologous species systems must be evaluated with caution.

## Introduction

Human diseases could be treated by selective manipulation of pathways involved in their pathogenesis. Several druggable targets were defined in humans [1], [2] including steroid metabolizing enzymes like 17β-hydroxysteroid dehydrogenases (17β-HSDs) controlling the biological potency of steroid hormones by redox reactions at position 17 of the steroid scaffold [3], [4], [5], [6], [7]. 17β-HSDs belong to the short-chain dehydrogenase/reductase superfamily (SDR) [8], except for 17β-HSD type 5 which is a member of aldoketoreductase (AKR) superfamily [9].

Since the observation of the prognostic value of 17β-HSDs in breast or prostate cancers [10], [11], [12], [13], [14] the research on these enzymes included development of specific inhibitors [15], [16], [17], [18], [19], [20], [21], [22], [23]. It was assumed that in hormone-dependent cancers an inhibitor of conversion of estrone to estradiol by 17β-HSD 1 would deplete the biologically active hormone estradiol from the signal transduction pathway and by that constrain cell proliferation in breast cancer or endometriosis. Therefore, extensive strategies included 17β-HSD 1 as a drug target [21], [22]. We recently contributed to this field by a development of novel effective inhibitors of this enzyme by exploring modifications at positions 2 or 15 of estrone (compounds **1**, **2** and **3** in this study) [24] and designing fluorine derivatives of estrone [25].

The growing number of genetically and functionally distinct 17β-HSDs makes it difficult to develop enzyme-specific inhibitors. At least fourteen types of 17β-HSDs are known so far with partly overlapping or reciprocal substrate preferences and not always distinct tissue distribution [5], [6], [7], [26], [27]. Furthermore, specificity analyses are affected by the nature of assay systems like *in vitro* assays with recombinant protein or *ex vivo* measurements in cell lines naturally expressing the enzyme. The verification of inhibition results seen for human 17β-HSDs in animal models, mostly rodents, has to cope with the problem of differences to humans in sex steroid metabolism [28], [29], [30], [31].

In this work we focused on the inhibition of 17β-HSDs converting estrogens and androgens. We analyzed (i) how susceptible human 17β-HSD 1, 2, 4, 5 and 7 were to inhibition by a novel class of 15-substituted estrogens described in our patents [24], and (ii) how the candidate inhibitors were modulating the activity of 17β-HSD 1 from different species including human, marmoset, pig, mouse and rat. Because profound differences between the orthologs in the susceptibility to inhibition were observed, we also analyzed (iii) if molecular docking experiments performed with modeled enzymes can differentiate or predict the efficacy of inhibitors.

## Results

### Validation of 17β-HSD Type Specificity

Several types of 17β-HSDs were chosen to check the specificity of recently developed inhibitors [24] against human 17b-HSD 1. Structure-function relationships were already reported for these inhibitors [24] and will not be analyzed in this manuscript. We monitored the inhibition at the physiological preferences of the 17β-HSDs, i.e. reduction of estrone to 17β-estradiol by 17β-HSD 1 and 7, the reduction of androstenedione to testosterone by 17β-HSD 5, and the oxidation of 17β-estradiol to estrone by types 2 and 4. We restricted our assay to this set of enzymes as they are active after recombinant expression in bacteria and could be used for fast, robust and inexpensive screens of inhibitors. Other 17β-HSD types require transfection into mammalian cell lines for activity assays (type 3 or 14, [32], [33]) or were excluded for being physiologically irrelevant to this study (type 12 [34]).

**Figure 1:**
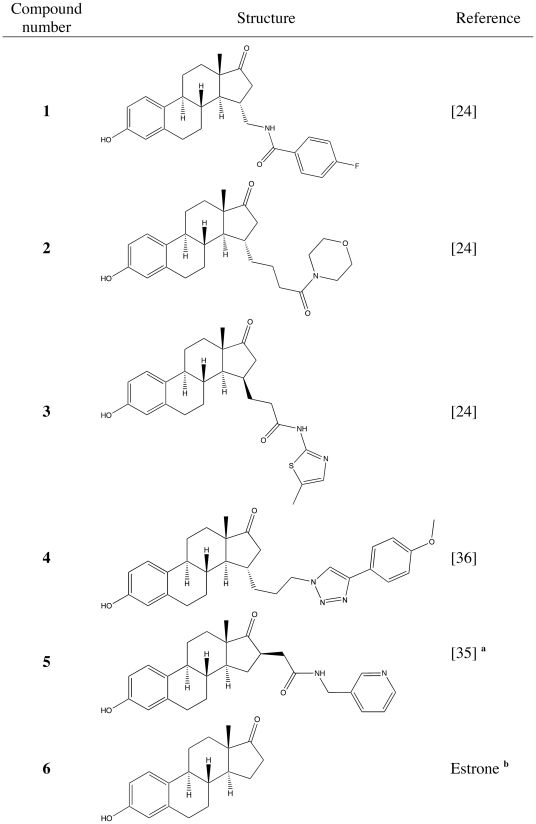
Structures of compounds used in the study. a Sterix compound, b product of estradiol oxidation.

**Figure 2:**
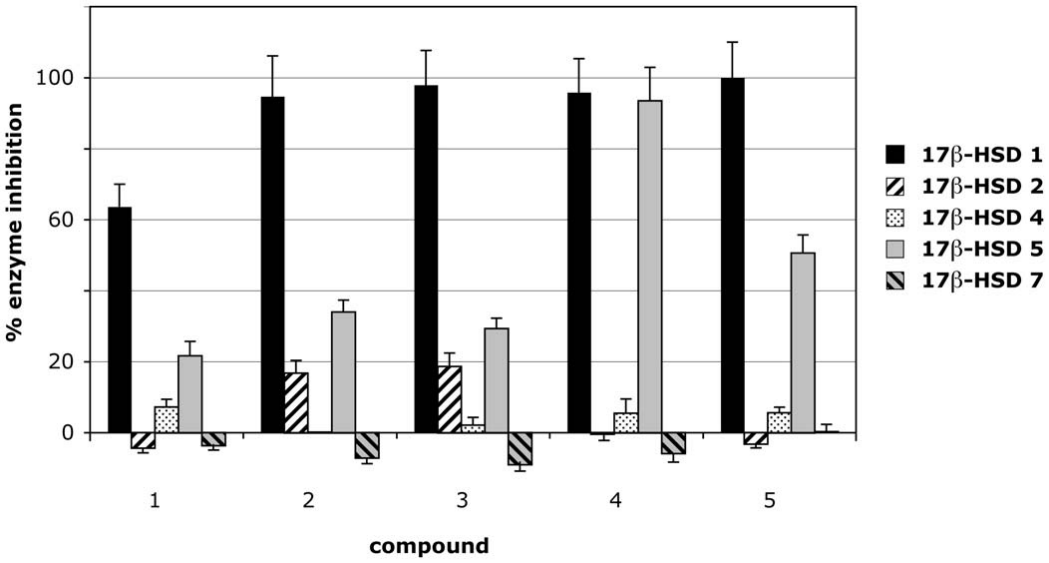
Analysis of inhibitor specificity among different human 17b-HSD types. Negative values correspond to a weak activation. All assays were run in duplicates of two independent experiments, normalized to control assay and reported as mean values ± SEM. Inhibitors were tested at 2 µM final concentration. Structures are shown in Figure 1. For referencing purposes the traditional and new names [8] of different human 17β-hydroxysteroid dehydrogenase types are given: 17β-HSD 1 – SDR28C1, 17β-HSD 2 – SDR9C2, 17β-HSD 4 – SDR8C1, 17β-HSD 5 – AKR1C3, 17β-HSD 7 – SDR37C1.

With this set of recombinant enzymes we have checked the relative inhibition of different reaction directions by 15-substituted estrogens [24] and a 16β-substituted estrogen [35] (for structures see Figure 1). We observed that compounds number **2** and **3** revealed high inhibition of the human 17β-HSD 1 reductive activity with very low inhibition of the other human 17β-HSDs (Figure 2). The substances reached a better selectivity than the Sterix reference compound **5** [35] especially showing less influence on 17β-HSD 5. However, as illustrated by measurements of our other compounds, not all substitutions at position 15 are very selective. For example substance **4** inhibits 17β-HSD 5 to the same amount as human 17β-HSD 1.

### Analysis of Inhibitor Influence on Activity of 17β-HSD 1 in Different Species

**Figure 3:**
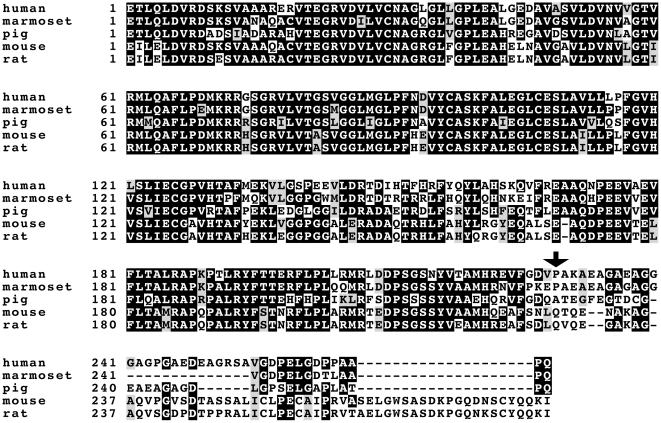
Amino acid sequence comparison of 17b-HSDs 1 of different species. Species are given to the left, followed by amino acid residue numbering. Identical amino acid residues are given white on black, whereas similar residues are grey shaded. Arrow points to the last C-terminal amino acid resolved in the crystal structure of PDB entry 1A27.

We have included all inhibitors in the next testing of susceptibility to inhibition of 17β-HSD 1 in different species. We prepared a set of recombinant 17β-HSDs 1 originating from human, marmoset, pig, mouse and rat. These 17β-HSD 1 enzymes reveal high level of amino acid similarity (Figure 3) ranging from 85% for human-marmoset to 78% for human-rat pairwise comparisons. The most divergent residues of the sequences are located in their C-terminal parts.

**Figure 4:**
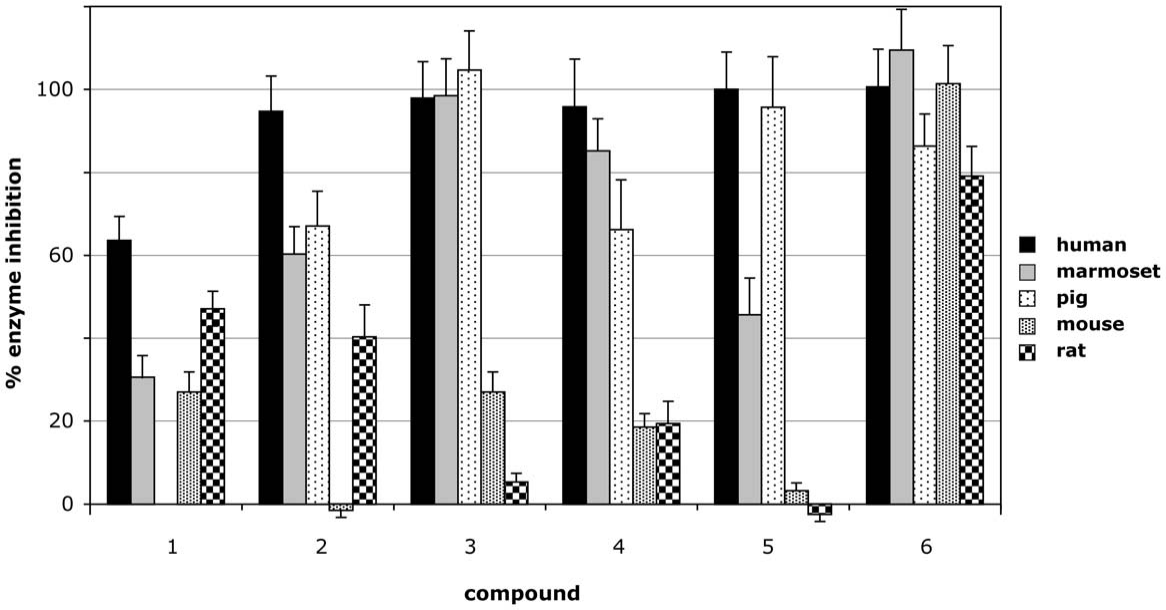
Inhibitors of activity of 17b-HSD 1 of different species. Different inhibitors were tested. Estrone (compound 6) was included to check the inhibition by the native substrate. Negative values correspond to a weak activation. All assays were run in duplicates of two independent experiments, normalized to control assay and mean values ± SEM reported. Inhibitors were tested at 2 µM final concentration. For structures see Figure 1.

Clear differences in the inhibitor influence on activity of 17β-HSD 1 of different species were observed (Figure 4). Surprisingly, the rodent enzymes revealed the biggest discrepancies to values measured for the human enzymes with all inhibitors. Comparable inhibition efficacy to that of human 17β-HSD 1 was observed for the marmoset and pig enzymes. To facilitate normalization and direct comparison with published records we included estrone (compound **6**) to our study. The estrone was used because it is a natural ligand of 17β-HSD 1 and because it causes substrate inhibition in higher concentrations by a formation of dead-end complex [37]. The estrone turned out to be a potent inhibitor of all tested 17β-HSD 1 orthologs.

We further checked the inhibition of the best inhibitors, the compounds **2** and **3**, by IC_50_ determination (Table 1). These data allowed quantification of efficacy of inhibition between 17β-HSDs 1 of different species. Pig 17β-HSD 1 is affected by both inhibitors in nearly the same concentration range as the human enzyme. Marmoset monkey 17β-HSD 1 requires a higher concentration to be blocked. The rodent enzymes were inhibited only up to 10 and 40% by compounds **2** and **3**, respectively. This precluded IC_50_ determination for the rodent enzymes.

**Table 1:** Comparison of IC50 values obtained for inhibition of 17b-HSDs 1 in different species.

Compound number	IC_50_				
	Human	Marmoset	Pig	Mouse	Rat
**2**	87.4 nM	3607 uM	457 nM	nd	nd
**3**	1.3 nM	95.3 nM	0.34 nM	nd	nd

nd - not to determine, due to too low inhibition.

### Validation of Candidate Compounds by Molecular Docking

Although the overall amino acid sequences of 17β-HSD 1 are very similar in different species (Figure 3) some differences are present. These differences lead to structural changes in enzyme substrate binding pocket and therefore add to differences in the potency of inhibitors in the different enzymes. We checked if molecular docking experiments can contribute to the challenge of prediction of inhibitor specificity. Molecular docking is a valuable approach in the analyses of ligand-protein interaction and can be used for pre-selection of pharmacophores as candidates for enzyme inhibitors. To accomplish that we performed docking of inhibitors to models of the different 17β-HSDs that were also enzymatically tested in this study. Please note that the docking experiments were performed only including most similar parts of enzymes, i.e. taking the amino acid sequence from the N-terminus up to the position marked by an arrow as shown in the Figure 3. The divergent C-terminal parts of proteins were neither used in modeling nor docking studies.

We first performed a global comparison of all enzyme types in all species with known *in vitro* inhibition data. Results of the first round of docking experiments are depicted in the Supporting Information (Table S1). Experimental inhibition effects were available for 49 protein-compound pairs. The absolute correlation between scores predicted by 7 docking programs and measured inhibition ranged between 1% and 36% (AutoDock: 36%, eHits: 26%, Cdocker: 19%, SurFlex: 16%, Dock: 12%, LigFit: 9%, Glide: 1%). Higher docking scores correspond to higher fitting of compounds into the protein structures.

**Figure 5:**
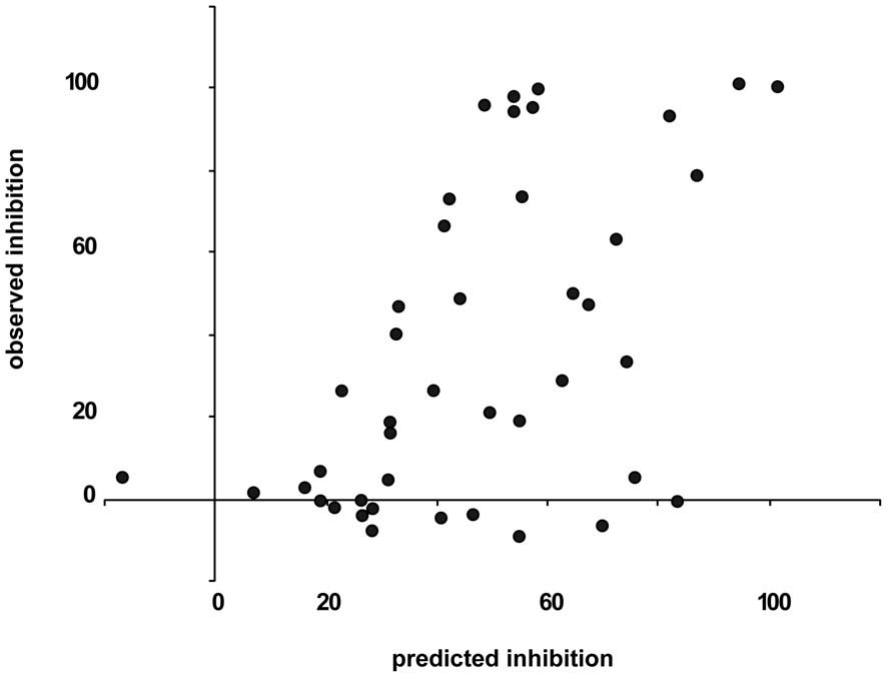
Relation between the predicted and observed inhibition values. Results are given for a modified consensus method that utilizes only two docking programs AutoDock and eHits.

We realized that the correlation in this set of protein-compound pairs should not be used to judge the quality of the docking programs. This is because the correlation varies a lot due to flexibility of both the protein and the compound. However, when applying a consensus mode instead of individual approaches an assessment of 17β-HSD inhibitors can be gained. When exploring a consensus of the docking methods we observed a correlation of 57%. However, when the jackknife procedure for elimination of training (memorization) effects was employed the correlation coefficient dropped to 32%, which is lower than the best performing method on this set (AutoDock: 36%). A modified consensus method that utilizes only 2 docking programs (AutoDock & eHits) exhibited an improved correlation to 41%, which was better than any single docking method in the set. Only this final method was used for subsequent data analyses. The relation between all predicted and measured inhibition values is visualized in Figure 5.

Next we analyzed data from 17β-HSDs 1 of different species. For human and marmoset data there is a good correlation between the predicted ranking of compounds as inhibitors by molecular docking scores and measured inhibition efficacy (Table 2). Porcine and rodent enzymes show much less susceptibility to inhibitors and their measured inhibition values reveal less correlation with the corresponding docking scores. For all 5 species models of the 17β-HSD 1 enzymes the consensus method predicted estrone (compound **6**) as the most potent inhibitor (best fitting compound) in agreement with the experimental data (Figure 4 and Table 2). On the average, estrone is the best natural inhibitor for all species studied.

**Table 2:** Comparison of predicted and measured inhibition for 17b-HSD 1 orthologs of five species.

Predicted consensus score						
Species	Compound					
	1	2	3	4	5	6
Human	72.1	53.6	53.6	57.1	58.1	101.3
Marmoset	38.1	45.2	52.5	58.5	35.7	78.1
Mouse	39.2	21.3	22.4	57.1	15.8	94.3
Pig	n.m.	−1.5	24.0	35.0	35.3	74.6
Rat	32.9	32.3	30.9	54.8	27.8	86.7
**average:**	**45.6**	**30.2**	**36.7**	**52.5**	**34.5**	**87.0**

Observed % inhibition						
Species	Compound					
	1	2	3	4	5	6
Human	63.6	94.7	97.9	95.8	100.0	100.6
Marmoset	30.6	60.3	98.5	85.2	45.6	109.5
Mouse	27.0	−1.5	27.0	18.5	3.2	101.4
Pig	n.m.	67.0	104.7	66.2	95.7	86.3
Rat	47.1	40.3	5.3	19.4	−2.4	79.1
**average:**	**42.1**	**52.2**	**66.7**	**57.0**	**48.4**	**95.4**

Predicted values correspond to molecular docking scores, observed values are from enzymatic assays at 2 µM compound concentration, n.m. – not measured.

We further analyzed the predictive value of molecular docking for different human 17β-HSD types. This docking approach ranked compounds **1** and **2** as best *in silico* hits. However, the measured inhibition ranks compounds **2**, **3** and **1** as most specific inhibitors of human 17β-HSDs 1. This observation is based on the lowest inhibition for the human 17β-HSD 2, 4, 5, and 7 at highest inhibition of 17β-HSDs 1 (Table 3).

**Table 3:** Comparison of predicted and observed inhibition for five compounds and four human 17β-HSD 1 homologs.

Predicted consensus score					
17β-HSD type_speciesa^a^	Compound				
	1	2	3	4	5
1_human	72.1	53.6	53.6	57.1	58.1
2_human	40.3	31.3	31.5	83.0	26.5
4_human	18.6	18.7	6.6	75.7	−17.1
5_human	49.3	73.9	62.6	81.7	64.5
7_human	46.3	27.9	54.7	69.5	25.9

Observed % inhibition					
17β-HSD type_species^a^	Compound				
	1	2	3	4	5
1_human	63.6	94.7	97.9	95.8	100.0
2_human	−4.3	16.7	18.6	−0.3	−3.2
4_human	7.2	0.1	2.1	5.5	5.6
5_human	21.6	33.9	29.3	93.5	50.6
7_human	−3.6	−7.1	−9.0	−5.8	0.0

Predicted values correspond to molecular docking scores, observed values are from enzymatic assays at 2 µM compound concentration.

a type of enzyme is separated by underscore from species description.

## Discussion

### Rationale for Inhibitor Search

The development of therapies for estrogen-dependent human diseases addresses the pre-receptor metabolism [38], [39], [40], which includes inhibition of enzymes like steroid sulfatase (STS), P450 aromatase and 17β-HSD 1. The design and application of STS inhibitors [41], [42], aromatase inhibitors [43], [44], [45], and combined STS-aromatase inhibitors [46] are showing significant therapeutic promise. On the other hand, the inhibitors for human 17β-HSD 1 are still at an early stage of development [34], [47], [48], [49] and have not reached clinical studies yet. Nevertheless, many efforts were undertaken in the finding effective inhibitors for human 17β-HSD 1 [21]. Selective 17β-HSD 1 inhibitors were reported with modifications of the steroid scaffold at positions 6, 16 or 17 [16], [18], [19], [50], [51], [52], substitution with sulfamates [53], [54], benzenes [55] or fluorine [25], in form of hybrid inhibitors constituted of estradiol with adenosine [17], [56], [57] and non-steroidal compounds [58], [59], [60]. Their activities are already reaching effective and selective inhibition of the human 17β-HSD 1 with pharmacologically attractive IC_50_ values in the nanomolar range. Our recent patents on 15-substituted estrone [24], [61] contributed to a new direction to this research.

### Challenge of Animal Models

Although animal models found broad applications in drug discovery they are not ideal phenocopies of human physiology in health and disease. Both enzyme expression levels and amino acid compositions of homologous enzymes are not the same. Consequently substrate preferences of steroid metabolizing enzymes in humans and other mammalian species are different for estrogens, androgens and glucocorticoids [7], [29] and in turn drug susceptibility is expected also to be different. Recently, inhibitors of glucocorticoid metabolism were shown to effect orthologs of different mammalian species [62] to various extent. Similar experiments including several species at the same time were not yet performed. Only one publication addressed the inhibitory potency of putative drugs against estrogenic 17β-HSDs in rats [31].

Our results now prove that the estrogenic 17β-HSDs 1 from different species indeed are distinctly affected by inhibitory compounds. Especially the lack of inhibition of the rodent enzymes by the most potent inhibitors of human 17β-HSD 1 is to be underlined. This is not very surprising since it is well known that rodent steroid metabolism differs from that in humans [28], [29], [30]. However, we provide a ranking of inhibitor efficacy for enzymes in different species. In case of preclinical animal tests, which are usually performed in mouse or rat, the most potent inhibitory compounds would have been sorted out before entering further development for human application.

### Lessons from Docking Experiments

Several novel potential inhibitors for 17β-hydroxysteroid dehydrogenases have been docked using available algorithms but applying a novel set of auxiliary simulation scripts. Although scoring accuracy and range of applications of computational docking has improved in the last years, resulting partially from increasing computing power, this method is far from excellence and still cannot be applied to practical tasks without *in vitro* and *in vivo* validation. Nevertheless, the method was able to confirm the choice of one of the two universal inhibitors and was able to select the most specific human 17β-HSD 1 inhibitor based on docking results on human 17β-HSD homologs, despite generally quite low correlation between the docking scores and observed inhibition. However, at present molecular modeling experiments done on modeled enzyme structures should be interpreted with caution.

### Closing Remarks

In this work we contributed to the field of inhibitor development in estrogen metabolism by 17β-HSD 1 by the quantification of inhibitor preferences between human and animal models used in the process of drug screening. Based on our data, steroid metabolism inhibitor development should be validated rather with primates or pig than with rodents. Otherwise, good candidate compounds against human targets would be already out-selected by experiments in the rodent model during pre-clinical optimization steps although they might have been specific and valuable drugs in disease treatment in humans.

## Materials and Methods

### Compound Synthesis

Compounds were synthesized as described elsewhere [24], compound **5** developed by Sterix (Ipsen SA) was re-synthesized according to [35]. Structures of compounds used for testing are given in Figure 1.

### Expression of Recombinant Enzymes in E.coli

Full length cDNAs of several 17β-HSDs type 1 originating from different species were cloned either into the pQE30 vector (human 17β-HSD 1, coding for acc. no. NP_000404) for expression as His-Tag protein or into a modified pGex-2T vector [63] (mouse 17β-HSD 1, acc. no. NP_034605; rat 17β-HSD 1, acc. no. NP_036983; marmoset 17β-HSD 1, acc. no. AAG01115; porcine 17β-HSD 1, acc. no. NP_001121944) for expression as GST-fusion proteins. The marmoset 17β-HSD 1 enzyme sequence was updated by the missing N-terminal part (AF272013) and the new porcine sequence was submitted to GenBank (NP_001121944). Human 17β-HSDs 2, 4, 5 (AKR1C3, the latter kindly provided by Dr. T. Penning) and 7 were all cloned into the modified pGEX-2T vector. For 17β-HSD 4 only the SDR-domain converting the steroids was subcloned [63]. Plasmids were transformed into *E.coli* BL21 DE3 Codon Plus RP (Stratagene) and enzyme expression was induced by 0.5 mM IPTG. After 4h incubation at 37°C with continuous shaking bacteria were pelleted by centrifugation at 10.000×g. Pellets were stored until use at −20°C.

### Enzyme Identities

Recently, the international SDR-Initiative has recommended [8] a new nomenclature for the human enzymes analyzed in this study. Here we provide for referencing purposes traditional and new names: 17β-HSD 1 – SDR28C1, 17β-HSD 2 – SDR9C2, 17β-HSD 4 – SDR8C1, 17β-HSD 5 – AKR1C3, 17β-HSD 7 – SDR37C1.

### In Vitro Measurement of Enzymatic Activity

Catalytic activity towards estrone and estradiol was assessed as originally described [25], [64] with minor modifications. The bacteria containing recombinant enzymes were resuspended in PBS and enzymatic assays were performed in 100 mM sodium phosphate buffer at pH 6.6 for the reductive reaction and at pH 7.7 for the oxidative reaction. The concentration of ^3^H-labelled steroid substrates in the reaction mixtures were 15 nM for estrone (2,4,6,7-^3^H(N)) in assays of 17β-HSD 1 and 7), 21 nM for estradiol (6,7-^3^H(N)) in assays of 17β-HSD 2 and 4, and 21 nM for androstenedione (1,2,6,7-^3^H(N)) in assays for 17β-HSD 5. All substrates were purchased from NEN/Perkin Elmer. The cofactors NADPH (Sigma; for reductive reactions) and NAD^+^ (Serva, for oxidative reactions) were used at final concentrations of 0.5 mg/ml. Potential inhibitors (dissolved in DMSO) were added in a final concentration of 2 µM or 0.005 µM to 5 µM in case of IC**_50_** determination (1% DMSO final each). The incubation at 37°C was stopped with 0.21 M ascorbic acid in methanol∶acetic acid 99∶1 (v∶v) after the time needed to convert approximately 30% of the substrate in a control assay with 1% DMSO, without inhibitor candidates. Substrates and products were extracted from the reaction mixture by SPE with Strata C18-E columns (Phenomenex), eluted by methanol and separated by RP-HPLC in a Beckman-Coulter system, using the column Luna 5 µm C18(2), 125×4.0 mm (Phenomenex). The solvent used was acetonitrile∶water (43∶57, v∶v) at a flow rate of 1 ml/min. Radioactivity was detected by online-scintillation counting (Berthold LB506D) after mixing with ReadyFlowIII (Beckman). Conversion was calculated from integration of substrate and product peaks. For calculation of inhibitory potential conversion of control assay (assay without inhibitor) was set to 0% inhibition. All assays were run in duplicates of two independent experiments and mean values are reported. The IC_50_ values were determined by the One Ligand Binding model of SigmaPlot kinetics module.

### Molecular Docking

The docking experiments where performed on 9 protein models, i.e. 17β-HSD 1 from human, marmoset, mouse, rat and pig, and further 17β-HSD 2, 4, 5 and 7 from human. Amino acid sequences were aligned with T-coffee [65] and inspected with Boxshade 3.21 (http://www.ch.embnet.org/software/BOX_form.html).

Models where based on the crystal structures deposited in the Protein Data Bank. For the human enzymes 17beta-HSD type 1, 4, and 5 the PDB entries 1A27, 1ZBQ, and 2FGB, respectively, were directly used [66], [67], [68], [69]. If crystal structures were not available, a homology modeling procedure based on aligning the sequence of the target protein with the sequence of the closest homolog deposited in PDB was applied. For 17beta-HSD1 of other species and human 17β-HSD 2 the template 1A27 was used, for 17β-HSD7 entry 1N5D served as template. C-terminal parts of the proteins analyzed revealed lower similarities and were not included in the model building. This local dissimilarity a typical effect of SDR-protein family already approached by us in modeling studies [70]. Models where generated automatically using the MODELLER program (modbase.compbio.ucsf.edu/ModWeb20-html/modweb.html).

Docking of compounds was performed using the following 7 docking programs: AutoDock, Cdocker, eHits, LigFit, Dock, Surflex and Glide accessible as described [71]. From each program one final score was selected as estimator of the fitness function and predictor for the experimental inhibition.

The consensus scoring method was based on multivariate linear regression analysis (least squares method) which assigns coefficients to each of the 7 docking programs to maximize the fitness between a linear combination of the 7 docking scores multiplied by the coefficients (predicted values) and the observed experimental inhibition (observed values). To eliminate the training (memorization) effect a jackknife procedure was employed. The regression analysis for a respective tested compound-protein pair was conducted in this case by using only values obtained for other compound-protein pairs (removing the tested pair from the dataset).

Additionally, a modified consensus method was created that used only scores and correlation coefficients of 2 docking programs (AutoDock and eHits) performing best on our dataset (exhibiting highest correlation between the predicted and observed values) by setting the docking scores of 5 docking methods (Cdocker, LigFit, Dock, Surflex, Glide) to 0.

The estimation of the accuracy of the docking protocol was based on the Pearson correlation coefficient between the predicted score and the observed inhibition. The estimation was conducted also separately for each model and each compound. When assessing the correlation for a protein model only compound-protein pairs with this protein were left in the dataset. Likewise, the correlation for a compound was calculated only on pairs with this compound.

### Gene Bank Submissions

The sequence of marmoset 17β-HSD was extended by the missing N-terminal part (AF272013) and the porcine sequence received acc. no NP_001121944.

## Supporting Information

Table S1 Prediction results for inhibitors of different human 17β-HSD types in different species.
(0.12 MB DOC)
