# Supplementary material for: Species Used for Drug Testing Reveal Different Inhibition Susceptibility for 17beta-Hydroxysteroid Dehydrogenase Type 1
Source: PLoS One. 2010 Jun 8;5(6):e10969. doi: 10.1371/journal.pone.0010969 (PMC2882332; doi:10.1371/journal.pone.0010969)
Supplement: Table S1 — Prediction results for inhibitors of different human 17β-HSD types in different species. (0.12 MB DOC) [file pone.0010969.s001.doc]

**Table S1.** Prediction results for inhibitors of different human 17β-HSD types in different species.

| **17β-HSD** | **compound** | **AutoDock** | **eHits** | **Cdocker** | **SurFlex** | **Dock** | **LigFit** | **Glide** | **no-jack** | **jack** | **jackAH** | **% Inh^b^** |
| --- | --- | --- | --- | --- | --- | --- | --- | --- | --- | --- | --- | --- |
| **type_species^a^** | |  |  |  |  |  |  |  |  |  |  |  |
| 1_human | 1 | -7.9 | 7.5 | 7.4 | 3.8 | -53.0 | 70.9 | -7.4 | 78.5 | 79.7 | 72.1 | 63.6 |
| 1_human | 2 | -8.8 | 7.2 | 16.3 | 3.3 | -55.6 | 67.4 | -8.0 | 74.8 | 65.7 | 53.6 | 94.7 |
| 1_human | 3 | -8.6 | 9.4 | 18.0 | 4.7 | -54.3 | 73.4 | -7.6 | 71.4 | 62.2 | 53.6 | 97.9 |
| 1_human | 4 | -7.0 | -4.6 | -18.6 | 1.9 | -46.2 | 55.0 | -7.8 | 67.9 | 60.4 | 57.1 | 95.8 |
| 1_human | 5 | -9.1 | 13.1 | 20.3 | 4.1 | -56.9 | 75.8 | -9.0 | 77.9 | 70.2 | 58.1 | 100.0 |
| 1_human | 6 | -5.1 | 6.7 | 9.9 | 3.1 | -33.3 | 51.3 | -8.3 | 86.2 | 78.4 | 101.3 | 100.6 |
| 2_human | 1 | -8.8 | -3.5 | 11.1 | 3.1 | -46.1 | 64.2 | -7.5 | 27.4 | 29.1 | 40.3 | -4.3 |
| 2_human | 2 | -8.8 | -3.4 | 15.3 | 4.5 | -48.4 | 60.5 | -7.3 | 24.2 | 24.6 | 31.3 | 16.7 |
| 2_human | 3 | -8.8 | -3.1 | 13.5 | 5.2 | -51.3 | 72.3 | -7.6 | 25.7 | 28.8 | 31.5 | 18.6 |
| 2_human | 4 | -6.3 | -4.1 | -13.0 | 3.8 | -35.9 | 45.4 | -7.1 | 46.8 | 50.0 | 83.0 | -0.3 |
| 2_human | 5 | -8.8 | -4.5 | 21.3 | 6.0 | -52.3 | 73.4 | -7.9 | 35.8 | 24.7 | 26.5 | -3.2 |
| 4_human | 1 | -10.2 | -3.0 | 10.0 | 0.1 | -47.3 | 60.1 | -7.9 | 25.7 | 26.7 | 18.6 | 7.2 |
| 4_human | 2 | -9.9 | -4.0 | 11.4 | 2.4 | -54.3 | 67.5 | -6.4 | 22.6 | 27.1 | 18.7 | 0.1 |
| 4_human | 3 | -10.1 | -5.4 | 14.8 | 3.8 | -50.3 | 78.7 | -9.3 | 13.9 | 11.3 | 6.6 | 2.1 |
| 4_human | 4 | -5.8 | -2.9 | -36.2 | 4.6 | -40.6 | 31.1 | -7.1 | 60.4 | 75.1 | 75.7 | 5.5 |
| 4_human | 5 | -11.4 | -5.5 | 34.5 | 4.8 | -55.5 | 85.0 | -9.3 | -5.4 | -10.5 | -17.1 | 5.6 |
| 5_human | 1 | -6.8 | 0.8 | 20.0 | 3.9 | -52.9 | 80.2 | -8.7 | 81.4 | 84.3 | 49.3 | 21.6 |
| 5_human | 2 | -6.9 | 2.4 | 20.6 | 7.3 | -51.9 | 73.8 | -6.5 | 69.7 | 64.4 | 73.9 | 33.9 |
| 5_human | 3 | -7.0 | 0.3 | 18.0 | 4.0 | -55.5 | 79.0 | -6.1 | 69.1 | 82.9 | 62.6 | 29.3 |
| 5_human | 4 | -4.3 | -5.7 | -18.4 | 4.8 | -33.0 | 30.3 | -6.1 | 66.3 | 53.9 | 81.7 | 93.5 |
| 5_human | 5 | -6.8 | 0.8 | 28.6 | 7.3 | -51.9 | 84.0 | -9.7 | 49.4 | 64.7 | 64.5 | 50.6 |
| 7_human | 1 | -8.9 | -1.4 | 18.5 | 4.2 | -43.1 | 61.2 | -7.0 | 19.6 | 19.7 | 46.3 | -3.6 |
| 7_human | 2 | -9.7 | -1.7 | 18.8 | 6.0 | -47.9 | 57.0 | -6.0 | 12.4 | 5.9 | 28.0 | -7.1 |
| 7_human | 3 | -8.5 | -1.6 | 19.5 | 5.4 | -44.9 | 63.1 | -6.4 | 15.0 | 23.3 | 54.7 | -9.0 |
| 7_human | 4 | -6.0 | -3.7 | -21.8 | 3.8 | -43.2 | 63.5 | -7.9 | 63.8 | 76.2 | 69.5 | -5.8 |
| 7_human | 5 | -9.5 | -0.9 | 22.8 | 4.9 | -51.8 | 74.5 | -9.0 | 31.2 | 28.9 | 26.0 | 0.0 |
| 1_rat | 1 | -8.9 | -1.3 | 9.5 | 3.8 | -52.1 | 71.7 | -7.9 | 40.0 | 39.9 | 33.0 | 47.1 |
| 1_rat | 2 | -8.9 | -1.3 | 15.9 | 3.8 | -53.1 | 69.2 | -7.1 | 30.4 | 38.0 | 32.3 | 40.3 |
| 1_rat | 3 | -8.7 | -2.4 | 17.0 | 6.2 | -53.8 | 71.4 | -7.9 | 25.3 | 34.5 | 31.0 | 5.3 |
| 1_rat | 4 | -7.0 | -5.4 | -14.9 | 4.8 | -39.0 | -6.0 | -5.2 | 26.9 | 32.2 | 54.8 | 19.4 |
| 1_rat | 5 | -8.4 | -1.4 | 25.8 | 6.0 | -56.2 | 67.8 | -7.7 | 31.8 | 46.8 | 27.8 | -2.4 |
| 1_rat | 6 | -4.9 | -3.4 | 5.6 | 3.1 | -35.6 | 44.3 | -7.6 | 66.9 | 65.0 | 86.7 | 79.1 |
| 1_mouse | 1 | -8.0 | -3.6 | 7.8 | 2.9 | -50.1 | 52.1 | -7.6 | 48.2 | 50.0 | 39.2 | 27.0 |
| 1_mouse | 2 | -9.2 | -1.8 | 14.9 | 1.0 | -55.9 | 54.4 | -7.0 | 51.2 | 59.2 | 21.3 | -1.5 |
| 1_mouse | 3 | -9.2 | -3.0 | 12.6 | 3.0 | -56.6 | 61.4 | -7.2 | 33.8 | 42.9 | 22.5 | 27.0 |
| 1_mouse | 4 | -6.6 | -4.3 | -23.0 | 3.8 | -41.9 | -46.1 | -4.5 | 39.0 | 63.7 | 57.1 | 18.5 |
| 1_mouse | 5 | -9.5 | -1.8 | 25.6 | 5.6 | -56.4 | 75.0 | -8.9 | 29.2 | 31.8 | 15.8 | 3.2 |
| 1_mouse | 6 | -4.7 | -2.4 | 8.9 | 2.3 | -37.3 | 37.0 | -7.9 | 78.0 | 74.2 | 94.3 | 101.4 |
| 1_marmoset | 1 | -7.9 | -3.5 | 13.6 | 4.8 | -54.3 | 71.6 | -7.9 | 58.3 | 51.4 | 38.1 | 30.6 |
| 1_marmoset | 2 | -8.5 | -3.9 | 16.2 | 6.8 | -58.4 | 70.9 | -8.4 | 49.8 | 37.1 | 45.2 | 60.3 |
| 1_marmoset | 3 | -8.9 | -4.9 | 21.6 | 5.2 | -56.5 | 76.9 | -8.9 | 36.1 | 27.3 | 52.5 | 98.5 |
| 1_marmoset | 4 | -6.8 | -5.5 | -9.5 | 2.2 | -46.0 | 37.7 | -7.1 | 47.9 | 58.4 | 58.5 | 85.2 |
| 1_marmoset | 5 | -8.5 | -4.0 | 27.8 | 5.1 | -54.8 | 71.8 | -8.0 | 28.6 | 35.6 | 35.7 | 45.6 |
| 1_marmoset | 6 | -4.9 | -4.2 | 3.5 | 4.0 | -34.9 | 41.1 | -7.3 | 57.3 | 53.8 | 78.1 | 109.5 |
| 1_pig | 2 | -9.5 | -3.9 | 14.5 | 6.2 | -53.4 | 60.2 | -5.7 | 14.1 | 2.6 | -1.5 | 67.0 |
| 1_pig | 3 | -9.7 | -4.0 | 17.4 | 2.4 | -59.6 | 66.0 | -6.5 | 40.4 | 27.8 | 24.0 | 104.7 |
| 1_pig | 4 | -8.7 | -4.4 | -85.3 | 2.6 | -47.6 | -4.0 | -8.1 | 53.8 | 39.5 | 35.0 | 66.2 |
| 1_pig | 5 | -8.7 | -2.9 | 29.0 | 6.3 | -54.7 | 76.8 | -7.3 | 40.8 | 23.0 | 35.3 | 95.7 |
| 1_pig | 6 | -5.1 | -3.4 | 8.4 | 4.5 | -34.3 | 50.2 | -7.0 | 46.3 | 49.0 | 74.6 | 86.3 |
|  | **correlation:** | **0.36** | **0.26** | **-0.19** | **-0.16** | **0.12** | **-0.09** | **-0.01** | **0.57** | **0.32** | **0.41** |  |

Molecular docking scores are given for different algorithms applied.

**^a^** type of enzyme is separated by underscore from species description

**^b^** % of substrate conversion inhibition at 2µM compound concentration, values are taken from *in vitro* measurements.
